# Nested Levels of Adaptive Divergence: The Genetic Basis of Craniofacial Divergence and Ecological Sexual Dimorphism

**DOI:** 10.1534/g3.115.018226

**Published:** 2015-06-01

**Authors:** Kevin J. Parsons, Jason Wang, Graeme Anderson, R. Craig Albertson

**Affiliations:** * Institute of Biodiversity, Animal Health & Comparative Medicine, University of Glasgow, Glasgow G12 8QQ, United Kingdom; † Department of Biology, Morrill Science Center, University of Massachusetts, Amherst, Massachusetts 01003

**Keywords:** adaptive radiation, craniofacial, morphometrics, sexual antagonism, QTL mapping cichlid

## Abstract

Exemplary systems for adaptive divergence are often characterized by their large degrees of phenotypic variation. This variation represents the outcome of generations of diversifying selection. However, adaptive radiations can also contain a hierarchy of differentiation nested within them where species display only subtle phenotypic differences that still have substantial effects on ecology, function, and ultimately fitness. Sexual dimorphisms are also common in species displaying adaptive divergence and can be the result of differential selection between sexes that produce ecological differences between sexes. Understanding the genetic basis of subtle variation (between certain species or sexes) is therefore important for understanding the process of adaptive divergence. Using cichlids from the dramatic adaptive radiation of Lake Malawi, we focus on understanding the genetic basis of two aspects of relatively subtle phenotypic variation. This included a morphometric comparison of the patterns of craniofacial divergence between two ecologically similar species in relation to the larger adaptive radiation of Malawi, and male–female morphological divergence between their F_2_ hybrids. We then genetically map craniofacial traits within the context of sex and locate several regions of the genome that contribute to variation in craniofacial shape that is relevant to sexual dimorphism within species and subtle divergence between closely related species, and possibly to craniofacial divergence in the Malawi radiation as a whole. To enhance our search for candidate genes we take advantage of population genomic data and a genetic map that is anchored to the cichlid genome to determine which genes within our QTL regions are associated with SNPs that are alternatively fixed between species. This study provides a holistic understanding of the genetic underpinnings of adaptive divergence in craniofacial shape.

How particular changes in morphology relate to ecological diversification is a highly active area of investigation (Svanback and Eklov 2003; Cooper *et al.* 2010; Parsons *et al.* 2012; Praebel *et al.* 2013). Of particular interest are cases of rapid adaptive diversification, because they can often provide clear links between form and ecology (Robinson *et al.* 1996; Bolnick and Lau 2008; Parsons and Albertson 2009). While characterizing major patterns of morphological change within adaptive radiations can provide an important step toward understanding how functional diversity arises, there are also fine scale levels of phenotypic variation within such radiations that warrant closer investigation. Such "nested’ levels of variation can exist between closely related species, between populations of the same species, or even between sexes in ways that all contribute substantially to ecological success (Robinson *et al.* 1996; Foster *et al.* 1998; Jonsson and Skulason 2000; Parsons and Robinson 2007; Paradis and Magnan 2005; Riopel *et al.* 2008; Cooper *et al.* 2010). In addition, because ecological performance and morphological divergence do not necessarily scale linearly across adaptive radiations, even seemingly small phenotypic differences can have profound implications for evolution (Gray *et al.* 2005; Parsons and Robinson 2006; Parsons *et al.* 2011a). Therefore, investigating how such nested levels of variation may accumulate to contribute to larger patterns of divergence may be the key for understanding adaptive radiations. Specifically, understanding mechanisms that underlie nested levels of variation could provide a deeper understanding of how ecomorphological divergence within or between closely related species may precipitate wider adaptive radiations as a whole.

In line with the idea of nested phenotypic variation, magnitudes of adaptive divergence can vary across a common trajectory (Young *et al.* 2009; Cooper *et al.* 2010). For example, the cichlid fishes that inhabit the major lakes of East Africa’s rift valley are among the best known adaptive radiations. In a short amount of geological time (no more than 16 MY), Lakes Tanganyika, Malawi, and Victoria have fostered the explosive evolution of many hundreds of cichlid species (Turner *et al.* 2001). A recent comprehensive study across these cichlid radiations has determined that divergence in craniofacial morphology has happened in a largely stereotypical pattern (*i.e.*, along a shared trajectory) in all three radiations (Cooper *et al.* 2010, Parsons *et al.* 2011b). The major axis of craniofacial variation in all three radiations reflects divergence along a continuum between benthic–pelagic foraging behaviors. At the pelagic end of the spectrum are cichlids with relatively long oral jaws and shallow-sloping head profiles. The combination of these two traits orients the feeding apparatus toward the anterior tip of the head, which facilitates the capture of horizontally directed prey. At the other end of the spectrum are benthic species with relatively short jaws and steeply descending heads, which directs the jaws more ventrally to forage on material that is under the fish (Cooper *et al.* 2010, 2011b, and references therein). Notably, this repeated pattern of divergence occurs despite differences in the age of the radiation or magnitude of morphological change, which is consistent with the idea that nested variation has accumulated to a greater or lesser extent in radiations of different ages.

To date, nested patterns of variation have not been explicitly examined in a major adaptive radiation. In cichlids, fine-scale ecological differences are apparent within and among species that could be indicative of nested divergence whereby the magnitude of differences are relatively small but follow the same trajectory as the larger radiation (Streelman *et al.* 2007; Albertson 2008; Cooper *et al.* 2010). For example, while multiple species on the "biting" end of the spectrum graze on algae and possess short, stout jaws, they use different foraging tactics and subtle morphological differences to accommodate this task (Ribbink *et al.* 1983; Albertson 2008). In Lake Malawi this fine-scale niche segregation is apparent between *Labeotropheus fuelborni* (LF), which has a very broad mouth for scraping algae off the surface of rocks, and *Tropheops* "red cheek," which has a very narrow mouth for nipping longer threads of algae from rocks. At a functional level these strategies lead to different biomechanical loadings being imposed on the craniofacial skeleton (K. Parsons unpublished data). The foraging tactic of LF is likely to depend almost exclusively on "forward pressure" to gain leverage for scraping, whereas TRC is likely to experience a combination of forward, backward, and sideways forces as it pushes into rocks to take hold of algae and then simultaneously tugs and twists to rip threads of algae. These different foraging strategies are associated with subtle but consistent differences in craniofacial architecture in the lateral view, with LF possessing a more “extreme” biting morphology characterized by relatively shorter jaws and a steeper profile on average compared to TRC (Cooper *et al.* 2010; Parsons *et al.* 2014).

In addition to fine-scale interspecific differentiation, sexual dimorphism driven by ecological specialization (rather than sexual selection) can provide another nested level of divergence (Temeles *et al.* 2000, 2010; Bolnick and Lau 2008; Herler *et al.* 2010). This is known as ecological sexual divergence, but currently it is unknown how it may match or contribute to trajectories of divergence in large adaptive radiations. In fact, its contribution to adaptive radiations is arguably in doubt as theory predicts that ecological divergence between sexes should be present in a way that is exclusive from differences between ecomorphs (Bolnick and Doebeli 2003). This is because conventional divergence between subpopulations should promote reproductive isolation between ecomorphs, whereas ecological sexual divergence still requires interbreeding between specialist phenotypes. Nonetheless, Cooper *et al.* (2011a) found sex-based differences in the craniofacial morphology of threespine sticklebacks (*Gasterosteus aculeatus*) that represented a greater magnitude of variation than differences between ecomorphs. Notably, the pattern of shape divergence between sexes mirrored the type of variation observed between ecomorphs, suggesting that the ecologically driven sexual dimorphisms can evolve to dissipate disruptive selection (Slatkin 1984; Bolnick and Doebeli 2003). Thus, it seems adaptive radiations could be due to a combination of many nested levels of divergence that accumulate, and perhaps complement each other, to produce larger patterns of divergence.

We suggest that such complementation of nested factors can be partially explained by the genetic architecture of traits. For example, some loci that affect shape may be sex-linked (Parnell *et al.* 2012), which in turn would only allow these loci to be "seen" by selection intermittently across generations. In other words, some alleles may only adaptively evolve when present within a specific sex, but when found in the reciprocal sex they are effectively neutral or "hidden" from selection (Gibson and Dworkin 2004; Dworkin 2005; Kuttner *et al.* 2014). Such a scenario would permit ecological divergence to occur both among populations and between sexes. As such, we predict that sex will influence the genetic architecture of ecologically relevant craniofacial variation. In terms of interspecific variation, we predict that LF and TRC alleles will contribute to different aspects of craniofacial shape, and that LF alleles will contribute to an overall steeper profile and shorter jaws. We test these predictions using a hybrid cross between LF and TRC with the overarching goal to determine the genetic basis of fine-scale niche differences and the genetic basis of sexual dimorphism in craniofacial shape. We also aim to determine whether trajectories of fine-scale craniofacial divergence and sexual dimorphism match with larger patterns of divergence across the Malawi radiation. If trajectories match, then this could lend new weight to the idea that nested variation contributes to larger patterns of divergence. In all, these data will lead to a novel, complementary understanding of the genetic architecture of ecologically relevant craniofacial divergence between species and between sexes.

## Materials and Methods

### Study species and experimental cross

Details regarding species and husbandry are provided elsewhere (Parsons *et al.* 2011c; Brzozowski *et al.* 2012). In brief, we crossed two closely related Lake Malawi rock-dwelling cichlid species that differ subtly in lateral craniofacial profile, with LF possessing a more steeply shaped head. Individuals of *Labeotropheus fueleborni* were collected from Makanjila Point while TRC animals were collected from Chizumulu Island. A single wild-caught LF female was crossed to a single wild-caught TRC male. A full sibling F_1_ family was interbred to produce 268 F_2_ individuals for genetic mapping. F_2_ individuals were reared in 10-gallon glass aquaria for 1–2 months, and then in 40-gallon glass tanks for another 6–10 months. Because of space constraints, F_2_ families were often combined; however, no more than 35 individuals were ever raised in one tank and sex ratios were approximately balanced. Sex in the F_2_ was largely determined by external examination of pigmentation and vent size (much larger in females), and was verified via dissection of gonads in a small subset of animals (∼25).

### Morphometrics and shape analysis

**Figure 1:**
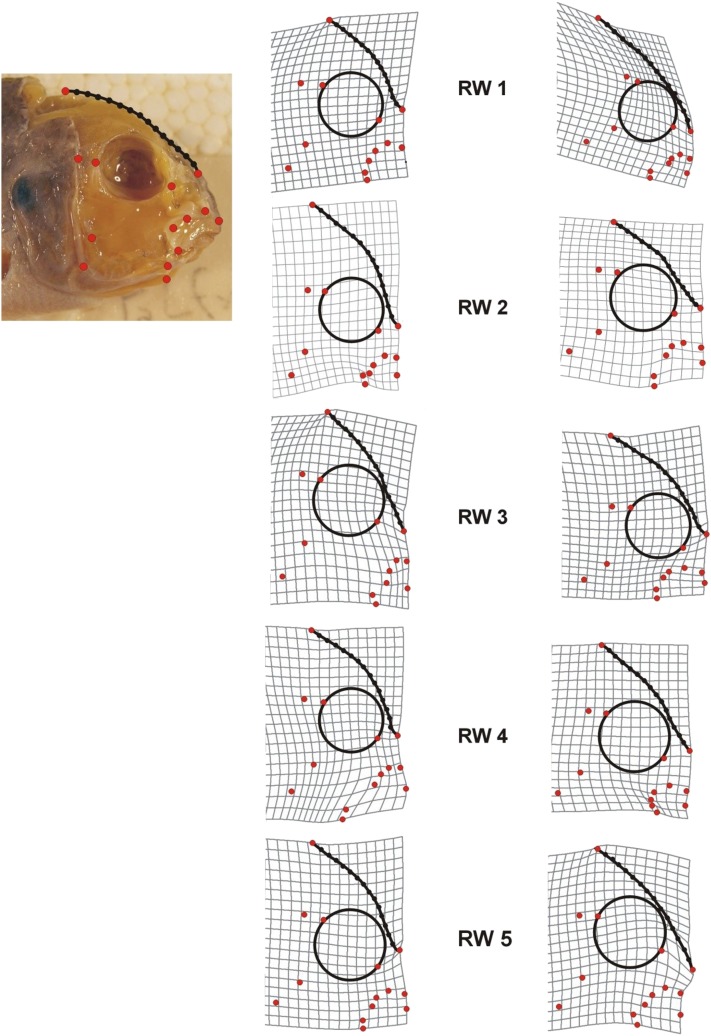
Landmarks, semilandmarks, and deformation grids depicting the first five axes of morphological variation [relative warps (RW)] in F_2_ hybrids. Across the different axes there are notable differences in craniofacial profile, most obviously in the cranial region quantified using semilandmarks (black dots and lines), but also with regard to the size of the preorbital region, size and positioning of the eye, and depth of the head.

Variation in the lateral view of the head in F_2_ hybrids was quantified using a geometric morphometric approach. A total of 14 regular homologous landmarks and 14 sliding semilandmarks were collected on the lateral view of the head (Figure 1). Sliding semilandmarks (Bookstein 1997) make possible the description of shapes combining curves and classic homologous landmarks on the same object, and the incorporation of these data has become standard in the field of morphometrics (Zimmerman *et al.* 2009; Arnegard *et al.* 2010). Semilandmarks made it possible to measure the profile of the head from the occipital crest to the dorsal tip of the ascending process of the premaxilla. Landmarks were superimposed by a generalized Procrustes superimposition (GPA) (Rohlf and Slice 1990), whereas semilandmarks were superimposed by allowing them to slide along curves bounded by landmarks to minimize the Procrustes distance among individuals (Bookstein 1997). To minimize the potential effects of allometry from the data we also performed a multiple regression of shape on geometric centroid size to generate landmark data sets based on residuals for further analysis. Landmark data were collected using TPSdig2, GPA was performed using Coordgen6h and multiple regression was performed using Standard6, whereas semilandmarks were slid using tpsRelw (all available at http://www.life.bio.sunysb.edu/morph/).

To generate data for statistical analysis we performed a thin-plate spline (TPS) procedure to generate partial warp scores. TPS models the form of an infinitely thin metal plate that is constrained at some combination of points (*i.e.*, landmarks) but is otherwise free to adopt a target form in a way that minimizes bending energy. In morphometrics, this interpolation is applied to a Cartesian coordinate system in which deformation grids are constructed from two landmark configurations (Bookstein 1991). The total deformation of the thin-plate spline can be decomposed into geometrically orthogonal components (partial warps) based on scale and used in multivariate statistics. To summarize multivariate data into orthogonal axes, we used a covariance-based principal components analysis [*i.e.*, relative warps (RWs)] using the software tpsRelw. Relative warps, which are the equivalent to principal components for geometric morphometric data, were then used as phenotypic variables in our QTL analysis.

However, relative warps do not necessarily relate to patterns of evolution. Therefore, to determine whether relative warps from F_2_s were relevant to the adaptive radiation of Lake Malawi cichlids as a whole, and to divergence between LF and TRC in particular, we compared their trajectories of variation (eigenvectors). Trajectories calculated from covariance matrices of F_2_s were compared to a preexisting dataset from Lake Malawi (Cooper *et al.* 2010) and to a combined dataset equally comprising the parental LF and TRC species (Parsons *et al.* 2011c). To facilitate this comparison, we collected additional semilandmark data as mentioned above. Landmark datasets from the F_2_s, parental species and the Malawi radiation were then appended and semilandmarks were slid in a common step, as was superimposition of regular landmarks. Comparisons of the trajectories between F_2_s and the Malawi radiation and between F_2_s and LF/TRC variation were made using 1600 runs of a bootstrapping procedure. The orientations of the first RW axes derived from each of the datasets were compared directly, but axes subsequent to RW1 could not be examined individually due to software limitations. All analyses that involved multiple axes determined whether the alignments of planes (when only two RW axes were examined) or multi-dimensional hyperplanes (“flat” surfaces of more than two dimensions embedded in larger dimensional spaces) were significantly different between two populations (*e.g.*, F_2_ *vs.* LF/TRC). When bootstrapping any two of the original sets of data, the sample sizes of the bootstrap sets produced from the larger of the two were the same as the sample sizes of both original datasets. Resampling the smaller of the original two datasets created two bootstrap sets of the same size as the original. These analyses were performed using the software package SpaceAngle36 (available at: http://www.life.bio.sunysb.edu/morph/).

Relative warps were also examined to determine whether they were impacted by sex. A one-way ANOVA using sex as a grouping variable was used to determine if the mean relative warp scores for a specific axis differed between sexes. Additionally, a multivariate test of differences in shape due to sex was performed on F_2_s using a discriminant function analysis (DFA) on partial warp scores (Zelditch *et al.* 2012). To represent the shape variation between sexes as deformation grids, we used regression of the canonical root scores derived from this model against landmark coordinates using the software TpsRegr (Rohlf 2011). To accentuate variation between sexes, deformation grids were magnified by a factor of three to aid in visual interpretation. The phenotypes of RWs 1–5, sex, and the DFA scores were used for subsequent QTL analysis.

### RAD-seq genotyping and population genomics

Although we follow a traditional F_2_ hybrid design for QTL mapping, we also genotyped individuals from natural populations to provide the ability to cross-reference loci of interest (*i.e.*, selection signature quantitative trait loci, hereafter ssQTL) (Parsons and Albertson 2013; Albertson *et al.* 2014). Although a relatively new terminology, ssQTL makes an explicit connection between loci identified as QTL in traditional laboratory crosses where genotype-phenotype relationships are much more tractable, and in wider population level patterns of genomic divergence where differentiation can be attributed to divergent natural selection. Given the largely homogenous genomes of Malawi cichlids (Loh *et al.* 2008), the finding of fixed or nearly fixed allelic differences between species is justified as a "signature" of such selection (*i.e.*, it is highly unlikely that these outlier loci are due to drift or other neutral processes). Although ssQTL are not conclusive about whether adaptive phenotypes are determined by these underlying loci, they can provide a highly informed and logical point of entry for functional genetic tests (Albertson *et al.* 2014). SNPs were identified across 268 F_2_ as well as 20 wild-caught LF from Makanjila Point and 20 wild-caught TRC from Chizumulu Island using restriction site–associated DNA sequencing (RAD-seq) (Miller *et al.* 2007). Genomic DNA was extracted from pectoral fin tissue using DNAeasy blood and tissue kits (Qiagen Inc. CA, USA), digested with the restriction enzyme *SbfI*, and processed into RAD libraries following the work of Chutimanitsakun *et al.* (2011). Bowtie (Langmead *et al.* 2009) was used to align reads to the reference cichlid sequence (*Metriaclima zebra* v.0), and SAMtools was used for SNP calling. In total, 42,724 SNPs were identified with a median sequencing depth of 33×. Most SNPs represented rare variants; thus, data were then filtered by F_ST_ values to include loci showing high differentiation between LF and TRC (F_ST_ ≥ 0.57, an empirical threshold for divergence between cichlid genera) (Mims *et al.* 2010) as well as deviations from Mendelian segregation in the F_2_. This resulted in 1395 loci. A complete list of SNPs with outlier F_ST_-values is provided elsewhere (Albertson *et al.* 2014).

### Linkage map construction and QTL analyses

Linkage map construction from our filtered dataset followed methods contained within the R/qtl package and are presented in detail specific to our cross within another work (Albertson *et al.* 2014). Briefly, the resulting map contained 948 loci consisting of 25 linkage groups, with 24 having between 13 and 76 loci each and one group containing 2 loci. The total map size was 1474.9 cM and linkage groups were numbered according to Lee *et al.* (2005).

The genetic architecture of craniofacial shape was characterized via several discrete QTL analyses: (1) a statistically liberal initial series of scans was used to identify putative loci and interactions that included all traits; (2) a more rigorous multiple QTL mapping (MQM) step was used to identify additional loci in the context of sex as a covariate; and (3) multiple QTL models were constructed that assessed the relative contributions of sex as well as loci and interactions identified in steps 1 and 2 in explaining phenotypic variation in the F_2_. For step 1, tests on the first five relative warp (*i.e.*, RW) scores, the previously mentioned canonical root scores from the DFA, and sex were conducted using R/qtl as described in Broman and Sen (2009). Sex was examined with standard interval mapping for bimodal traits, whereas QTL tests for relative warps and canonical root scores were performed using standard interval mapping for continuous traits. To identify QTL for relative warps while taking the effect of sex into account, a further model of standard interval mapping was also performed using sex as a covariate. Additionally, two-QTL scans on relative warps data were performed with sex as a covariate (epistatic interactions via Haley-Knott regressions). The putative QTL loci were identified as having a LOD score greater than 3 for standard interval mapping or a LOD score greater than the 95% threshold (created by 1000 permutations for a given model) for loci identified with two-QTL scans (following Miller *et al.* 2012). This collection of putative loci from all prior scans on relative warps was tested for verification by maximum likelihood–based backward elimination (*i.e.,* to specify cofactors) and permutation tests (*i.e.*, 95% threshold as determined by 1000 permutations) during subsequent rounds of MQM scans used for step 2 (Arends *et al.* 2010). In all of these MQM tests, the sex-determining locus was included in the model.

The final step in the QTL analysis (step 3) followed MQM methods presented in Broman and Sen (2009). Briefly, markers identified from steps 1 and 2 were included in these QTL models along with the sex phenotype. The logarithm of the odds (LOD) scores for interactions were calculated as the LOD for the full model minus the LOD for the additive model (Broman and Sen 2009). The variance accounted for by each QTL is calculated as 1 − 10^−(2/n)LOD^ (Broman and Sen 2009) and is reported as percent variance explained (PVE).

We consider step 1 as a preliminary analysis used to build more rigorous QTL models. These data are therefore presented as supplementary material (Supporting Information, Table S1). Results from steps 2 and 3 of our analysis are presented in the main text, because both offer a unique set of advantages when building QTL models. The MQM method of Arends *et al.* (2010) (step 2) is based on that of Jansen (1994), wherein unlinked QTL are used as cofactors in the analysis to more accurately detect and assess the effects of individual QTL. The method of Broman and Sen (step 3) is less powerful than that of Arends *et al.* (2010); however, it allows for greater flexibility in the construction of QTL models, such as the inclusion of interactions and sex as a phenotypic covariate.

Finally, we performed a test for interactions between loci identified for DFA scores (*i.e.*, sexual dimorphism in shape). The rationale here is that putatively autosomal markers may interact with the sex-determining locus to affect head shape in these fish. To test this, we examined loci identified from step 2 using the “addint” function in R/qtl.

### Depiction of shape QTL

To gain a general understanding of the impact of individual QTL loci on craniofacial shape, we modeled shape data against genotypic data using generalized linear models in TpsRegr (Rohlf 2011). This involved coding both types of homozygotes and heterozygous genotypes for the marker closest to a given QTL locus. We focused on loci with large effect sizes as determined by MQM analysis (step 2), and for which underlying SNPs showed exceptional levels of divergence between natural populations of LF and TRC at candidate loci for craniofacial development. This approach allowed us to more directly determine the impact of loci likely under the influence of selection on shape (ssQTL) (see Parsons and Albertson 2013). A regression was performed and differences were magnified by a factor of 10 to enhance our ability to interpret shape variation.

## Results

### Morphometrics: patterns of variation

The first five RW axes each explained more than 5% of the total variation in F_2_ shape and so became the focus of later analyses. Together they explained 57.5% of the variation (18.0%, 13.9%, 11.3%, 7.9%, and 6.4% explained variance for RWs 1–5, respectively) in craniofacial shape. Deformation grids depicting shape variation on each of the five RW axes suggested that notable differences in craniofacial profile were present, as were corresponding differences in the size of the preorbital region, size and positioning of the eye on the head, and length of the lower jaw (Figure 1).

**Figure 2:**
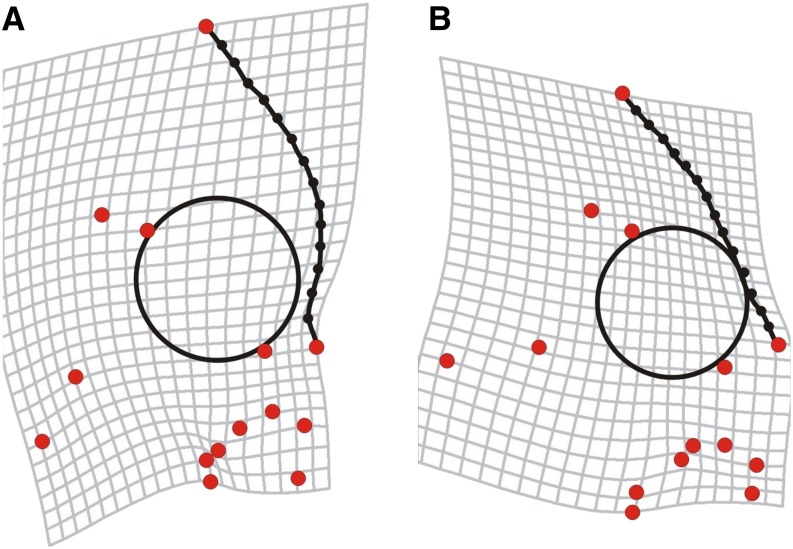
Morphological differences that occur between sexes within an F_2_ hybrid population of Tropheops sp. “red cheek” and Labeotropheus fueleborni. Males are depicted by (A) and have a steeper craniofacial profile, whereas females (B) have a more gradually sloping profile. Figures are created by regressing landmarks against discriminant function scores. Differences are magnified 3× to aid in interpretation.

Nested shape variation was supported by bootstrapping tests comparing the major trajectories of variation in the F_2_s to that of the Malawi radiation as a whole. These tests revealed that F_2_ shared their first major axis of variation with the Malawi radiation (*i.e.*, trajectories of RW 1s do not differ from random processes) (Table 1). Further, comparisons of eigenvector trajectories derived from F_2_s and a RW dataset derived from the combined data of parental species (LF and TRC) revealed that the first four axes of variation (*i.e.*, RWs 1–4) were also shared (Table 1). Thus, major trends in F_2_ shape are reflective of divergence between LF and TRC. Also, for three of the five axes (RWs 1, 2, 4), our ANOVA tests revealed that sex in the F_2_ had a significant effect on the variation of RW scores (Table 2). Sex was also found to have a significant impact on craniofacial shape in the F_2_ (*i.e.*, DFA, *P* < 0.01, 89% correct classification). Deformation grids depicting shape differences between sexes of the F_2_ hybrids showed that the profile of the head was the region with the greatest degree of variation (Figure 2). Males tended to have a more extreme biting morphology, including steeply sloped craniofacial profiles and changes in the position of muscle division points at the origin of the adductor mandibulae on the preoperculum.

**Table 1:** Comparative shape space orientations

Comparison	Observed Angle	Range of 95% C.I.s
LM *vs.* F2 RW1	58.5	0.12–69.88
F2 *vs.* LF/TRC RW1	75	0.02–82.57
F2 *vs.* LF/TRC RWs 1 and 2	70.7	0.96–85.30
F2 *vs.* LF/TRC RWs 1–3	83.9	1.81–86.08
F2 *vs.* LF/TRC RWs 1–4	67.0	2.53–94.94
F2 *vs.* LF/TRC RWs 1–5	100.5*	3.28–98.11

The range of bootstrap C.I.s for the observed angles between shape species (eigenvectors) were calculated by resampling data from both groups. Orientations were not considered significantly different if the observed angle fell within the range of C.I. values. Orientations that are significantly different are denoted with an asterisk

**Table 2:** Shape differences between sexes are significant

RW Axis	df	SS	Mean Sq	F	*P*
RW1	1	9.39	9.39	47.59	<0.001*
	173	34.12	0.20		
RW2	1	5.97	5.97	27.51	<0.001*
	173	37.54	0.22		
RW3	1	0.02	0.02	0.072	0.789
	173	43.49	0.25		
RW4	1	1.35	1.35	5.56	0.0195*
	173	42.15	0.24		
RW5	1	0.04	0.04	0.164	0.686
	173	43.47	0.25		

Here each relative warp was tested for differences using ANOVA with sex as a grouping variable. Significant differences are indicated by an asterisk beside their *P*.

Together these data suggest that the trajectory of our RWs, despite wide variation in scale, are relevant at multiple nested biological levels, including between sexes, between species, and across the Lake Malawi radiation as a whole. Similarly, the dissection of the genetic bases for these shape variables should have biological relevance across levels.

### QTL analyses: mapping sex and shape

Sex effects on shape were readily detectable in cichlid genetic architecture. A single qtl for sex explaining more than 33.8% of the variation was identified on linkage group (LG) 7 (Table 3). This locus is similar to that detected in another Lake Malawi F_2_ cross (Albertson *et al.* 2003), but not overlapping with a second major sex-determining locus on LG7 identified in a third Malawi cross (Parnell and Streelman 2013). Across the five relative warps, a total of 16 putative QTL (LOD > 3) were identified by models including sex as a covariate, and an additional four were identified by models including no sex covariate (Table S1). Two-QTL scan models with sex as a covariate identified one interaction each for RWs 2 and 5 (Table S1).

**Table 3:** Results of a genome scan for morphological QTL using multiple QTL models (MQM) based on the approach of Arends *et al.* (2010)

Trait	LOD*	LG	pos (cM)	Closest Marker	Interval (cM)	Phenotypic Score by Haplotype			Add	Dom	PVE (%)
						Lf/Lf	Lf/Trc	Trc/Trc			
RW1	7.52	7	15	c100.563038	12.9–24.7	−0.00754	−0.00259	0.01265	−0.00982	−0.00412	15.2
	4.54	3	15	c209.450133	5.0–23.5	−0.00698	−0.00008	0.00606	−0.00652	0.00042	9.5
	4.11	16	0	c96.18553	0.0–60.8	−0.00615	−0.00105	0.01124	−0.00870	−0.00307	8.6
RW2	9.26	7	5	c193.987462	0.0–35.0	−0.00914	−0.00094	0.01037	−0.00976	−0.00109	18.4
RW3	5.42	7	80	c89.778084	79.5–86.2	0.00548	−0.00373	0.00002	0.00273	−0.00461	11.2
	4.383	22	60	c259.89154	20.0–61.2	−0.00075	−0.00134	0.00309	−0.00192	−0.00184	9.2
	4.383	14	5	c158.620001	1.3–11.9	−0.00546	0.00309	−0.00108	−0.00219	0.00482	9.2
	3.591	5	35	c18.4038452	34.9–40.4	0.00306	−0.00270	0.00050	0.00128	−0.00313	7.6
	9.122	10.1	5	c22.6622454	0.0–5.0	−0.00365	−0.00201	0.00582	−0.00474	−0.00209	18.1
	3.967	19	5	c162.956970	0.9–16.5	0.00400	−0.00263	−0.00121	0.00260	−0.00271	8.3
RW4	3.91	13	30	c62.3129666	10.0–45.0	0.00598	−0.00267	−0.0019	0.003938	−0.00338	8.2
RW5	5.26	4	25	c61.3236042	17.4–30.0	0.00633	−0.00141	−0.00106	0.00369	−0.00334	10.9
	4.05	2	50	c11.4108123	0.0–52.7	0.00521	0.00023	−0.00385	0.00453	−0.00056	8.5
DF1 (sex)	23.19	7	30	c0.5071706	24.7–30.0	−1.29041	0.11693	1.50014	−2.04048	0.39865	39.9
	4.55	11	100	c207.450361	90.0–105.1	0.41163	−0.06853	−0.06784	0.23974	0.09504	9.5
	5.44	15	63.8	c74.200218	25.0–63.8	0.33120	0.23140	−0.97430	0.65275	0.11236	11.2

The effect of alleles on the traits (relative warp scores) are given. All LOD scores exceed the genome-wide 95% C.I. as determined by 1000 permutations of the data. RW, relative warp; DF1, discriminant function axis 1, which distinguishes F_2_ sexes; LG, linkage group; pos, position (cM); Add, additive effects; Dom, dominance effect; PVE, percent variance explained.

The MQM approach of Arends *et al.* (2010) (step 2) identified 13 QTL for RWs 1–5 that appear to be relatively evenly distributed across the genome. Notably, two QTL with the largest effect size for RW1 and RW2 colocalized with the sex-determining locus on LG7 (Table 3). Three additional loci were identified for sex-based differences in shape, as determined by DFA (*i.e.*, DF1) (Figure 2, Table 3), and here again the locus with the largest LOD score (23.2) mapped to the sex-determining locus on LG7. The other two QTL map to autosomal loci on LGs 11 and 15, but they were found to significantly interact with the sex-determining locus (Table 4).

**Table 4:** Interactions between the QTL associated with sex and QTL loci from other regions of the genome

QTL	df	Type III SS	LOD	% Var	F	*P* (chi^2^)	*P* (F)
7@30.0:11@100.0	4	26.19	2.41	2.8	0.025	0.03	0.03
7@30.0:15@63.8	4	30.99	2.86	3.3	0.011	0.01	0.01

Our final (step 3) MQM models were largely consistent with those from step two, although they also implicated additional craniofacial loci for RWs 1, 2, and 5. This approached yielded 14 QTL for RWs 1–5 that were distributed across the genome and one significant interaction for RW2. Consistent with our step 2 MQM analysis as well as our ANOVA tests of sex on RWs, we found that sex made a significant contribution to our QTL models for RWs 1 and 2. In addition, sex was found to contribute to the QTL model for RW4, which is also consistent with our ANOVA tests. Both our phenotypic and QTL analyses are consistent with the hypothesis that sex is an important factor in determining craniofacial shape.

### QTL analyses: allele effects

Allele effects for both MQM methods were generally consistent with our predictions. Positive scores on RWs 1 and 2 were associated with more shallow profiles and longer jaws, whereas negative scores were associated with steeper profiles and shorter jaws. For the more rigorous Arends MQM method, 4/4 QTLs for RWs 1 and 2 showed a pattern wherein the inheritance of TRC alleles was associated with positive RW scores (*e.g.*, shallow profile) and the LF alleles were associated with negative RW scores (*e.g.*, steep profile) (Table 3). These QTL also exhibited a largely additive mode of inheritance. Thus, LF alleles were associated with the development of a more extreme biting morphology along these two axes. Table 5 shows a similar pattern; however, this method also detected QTL of minor effect wherein LF alleles were associated with the development of positive RW1 and RW2 scores. This is not entirely unexpected because both LF and TRC have relatively steep craniofacial profiles, and our data suggest that different loci have been selected in each lineage to achieve this phenotype. RW3 explains variation in the overall depth of the skull, and especially the size of the upper jaws. There is no consistent pattern of effects for QTL that underlie this shape axis. RW4 describes variation in the length of the preopercle bone as well as the size and placement of the eye on the skull. Positive scores are associated with a long preopercle and large eye placed ventrally on the skull. The opercle bone series is part of a four-bar linkage model for lower jaw depression (Westneat 1990) and, all other things being equal, a longer set of opercle bones should translate to greater speed during jaw rotation (Hu and Albertson 2014). Combined with larger eyes that are placed lower on the skull, this configuration is consistent with a suction mode of feeding. TRC alleles are associated with the inheritance of this skull geometry. Although TRC is a biting specialist, other members of the *Tropheops* genus forage with a suction mode of feeding (Konings 2001; Albertson 2008). This allele may therefore contribute to variation along the biting–suction feeding ecomorphological axis in this lineage. Negative scores on RW4 are associated with a short preopercle and relatively small eye placed high on the skull. This configuration is consistent with a biting mode of feeding, and the LF allele contributes to the development of this skull type. RW5 deals with the placement of the eye on the skull as well as the size of the preorbital region of the skull. For each QTL, the LF allele is associated with positive RW5 scores; it is characterized by eyes placed relatively high on the head and a robust preorbital region of the skull. Alternatively, animals that inherit the TRC allele have eyes that are lower on the head and a greatly reduced preorbital region.

**Table 5:** Results of an MQM mapping procedure based on the method of Broman and Sen (2009) including sex as a cofactor

Trait	Model	LOD	PVE%	*P*	df	Closest Marker	Interval (cM)	Phenotypic Score by Haplotype		
								Lf/Lf	Lf/Trc	Trc/Trc
RW1	Full:	12.89	22.8	>0.0001						
	Drop-one:									
	LG1 @ 30.9 cM	1.72	3.3	0.0238	2	c7.4421711	7–61	0.00399	0.00037	−0.00553
	LG3 @ 8.2 cM	2.73	5.3	0.0027	2	c401.23855	2.1–35.0	−0.00754	0.00022	0.00585
	LG7 @ 14.6 cM	2.34	4.5	0.0063	2	c124.1667602	0–46.6	−0.00756	−0.00259	0.01264
	LG13 @ 0 cM	3.69	7.2	0.0003	2	c26.2061138	0–17	−0.00846	0.00154	0.00310
	Sex	1.33	2.5	0.0164	1					
RW2	Full:	18.5	38.5	>0.0001						
	Drop-one:									
	LG7 @ 46.6 cM	3.55	6.0	0.0172	6	c0.18725296	0–59	−0.01015	0.00025	0.00791
	LG8 @ 2.9 cM	2.82	4.7	0.0024	2	c106.1809812	0–55	0.00434	0.00061	−0.00943
	LG16 @ 24.1 cM	3.02	5.1	0.0414	6	c52.3743243	9–60.8	−0.00199	0.00099	−0.00098
	Sex	9.16	16.8	>0.0001	1					
	LG7 @ 46.6 cM × LG16 @ 24.1 cM	2.86	4.8	0.0149	4					
RW3	Full:	9.87	15.4	>0.0001						
	Drop-one:									
	LG7 @ 81.0 cM	2.63	5.0	0.0033	2	c32.5715629	75–84	0.00514	−0.00398	0.00072
	LG10 @ 2.0 cM	3.57	6.8	0.0004	2	c22.4015123	0–7.2	−0.00524	−0.00152	0.00627
	LG19 @ 16.5 cM	1.95	3.6	0.0143	2	c60.3623148	0–22.0	0.00316	−0.00119	−0.00254
RW4	Full:	3.20	8.6	0.0020						
	Drop-one:									
	LG13 @ 32.4 cM	2.10	5.2	0.0090	2	c0.5071706	0–45.0	−0.00243	−0.00137	0.00376
	Sex	1.37	3.4	0.0133	1					
RW5	Full:	7.91	14.2	>0.0001						
	Drop-one:									
	LG2 @ 49.7 cM	2.84	5.1	0.0018	2	c11.8641264	0–52.7	0.00546	0.00007	−0.00337
	LG4 @ 24.6 cM	2.94	5.5	0.0015	2	c28.74404	0–62.0	0.00662	−0.00144	−0.00106
	LG10 @ 0.0 cM	1.74	3.6	0.0210	2	c22.2628020	0–31.8	0.00478	−0.00069	−0.00201

Positions of QTL are given in centimorgans for each linkage group. The effect of alleles on shape traits (RWs) are given, with values of *Labeotropheus fueleborni* (Lf) and *Tropheops* "red cheek" (Trc) homozygotes and heterozygotes provided. RW, relative warp; LG, linkage group; PVE%, percent variance explained.

The QTL for DF1, which is the axis that distinguishes sex, exhibited a largely additive mode of action. For the major QTL on LG7, the allele effects were consistent with patterns of sex determination—*i.e.*, the TRC allele (male in this cross) led to positive DF1 scores and a male-like phenotype, whereas the LF allele (female in this cross) led to negative scores and a female-like phenotype (Figure 2, Table 3). In contrast, the two autosomal QTL showed a pattern wherein the LF alleles were associated with the development of more male-like skull morphology, and TRC alleles were associated with the development of more female skull morphology. Notably, both autosomal loci interact with the sex-linked locus (Table 4) such that the inheritance of two LF alleles at the autosomal QTL plus two TRC (male) alleles at the sex-linked locus leads to the development of male-specific craniofacial architecture. In other words, the LF alleles at these autosomal loci have the ability to “enhance” patterns of sexual dimorphism with respect to a biting morphology.

### Effects of individual QTL on craniofacial shape

**Figure 3:**
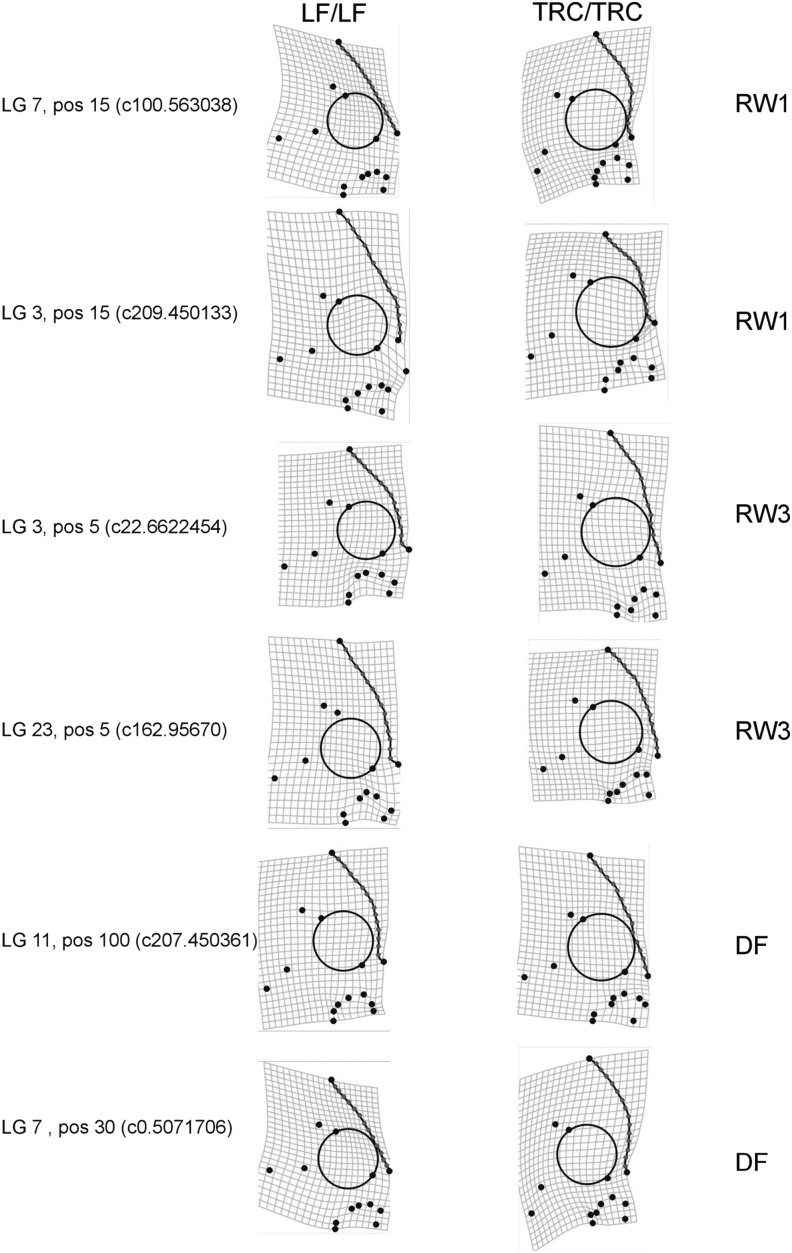
A depiction of the effect of QTL loci on craniofacial shape for a subset of selection signature QTL (ssQTL). Deformation grids on the left depict the average shape of individuals that are homozygous for *Labeotropheus fueleborni* alleles relative to individuals that are homozygous for *Tropheops* "red cheek" alleles on the right. The generalized linear models used to depict shape also include heterozygous genotypes. Allelic haplotypes are derived from the genetic marker that was mapped closest to the QTL peak (listed on the far left) linkage group number (LG) and chromosomal position (pos) in centimorgans are given. Specific traits (*e.g.*, RW1) for each QTL are given to the far right. Effects are also magnified 10× to facilitate visual interpretation.

To gain a more explicit idea of what aspects of shape variation were explained by individual QTL, we used a regression-based method to visualize the effect of the marker closest to the LOD peak of each QTL for craniofacial shape. Shape variation associated with alleles at these QTL showed a largely predictable pattern with regard to profile. Specifically, individuals homozygous for the LF allele usually had a steeper profile relative to individuals homozygous for the TRC allele (Figure 3). Head profile appeared to be "stepped" for individuals possessing the male or LF alleles, and the overall depth of the head was also more pronounced, particularly for QTL located on LGs 3 (position = 15 cM) and 23 (position = 5 cM) (QTL presented in Table 3).

## Discussion

### Ecomorphological patterns of variation are relevant at multiple levels

We have examined phenotypic variation at a fine scale by performing a cross between cichlids with relatively similar craniofacial profiles. However, despite the small differences in craniofacial anatomy relative to that of the Malawi adaptive radiation as a whole, our findings provide a direct inroad into the genetic basis of nested craniofacial variation that is relevant across multiple levels of organization.

Based on morphometric data, the primary axis of variation was shared across the radiation, between LF and TRC, and across our F_2_ hybrid population. This suggests that key aspects of the divergence between LF and TRC, and within our cross, are relevant to the adaptive radiation as a whole (Cooper *et al.* 2010; Parsons *et al.* 2011c). The similarity between closely related species and the radiation suggests that while the Lake Malawi radiation has been described as an "explosive" process, it relied on the accumulation of subtle differences across multiple subpopulations. These morphological findings should therefore enhance the importance of findings from our QTL analysis. In particular, we suggest that loci associated with the primary axis of variation in this cross may have played a role in distinguishing morphological species boundaries for LF and TRC, as well as distinguishing species across the radiation as a whole. If true, then such loci may also provide insight into the evolvability of craniofacial profile in this group because LF represents an extreme craniofacial phenotype across African rift lake cichlids (Cooper *et al.* 2010). Therefore, loci that contribute to this phenotype may be defining the limits of evolution for African cichlids (Parsons *et al.* 2014). Our F_ST_ data set provides some support for this assertion, with LF possessing the evolutionarily derived allele for many candidate loci within QTL intervals that exhibit outlier F_ST_ values. Thus, the extreme craniofacial shape exhibited by LF may be due to the accumulation of a unique combination of many alleles at craniofacial loci. Evolutionary divergence via the combined effects of many regions of the genome is emerging as a common theme in cichlids for a variety of traits (Albertson *et al.* 2014; Brawand *et al.* 2014; Loh *et al.* 2013). Verifying the causative loci that underlie these QTL and their evolutionary history will require further research.

The major trajectory of shape variation in this cross involved changes in craniofacial profile. At one end of the spectrum head profiles were steeper and almost "stepped" in appearance, whereas at the other end the profile they are more shallow and continuous. The oral jaws also showed a subtle but consistent lengthening along this axis such that animals with steeper profiles had shorter jaws, whereas those with more shallow profiles had relatively longer jaws. This pattern of covariation is consistent with that seen in another F_2_ population derived from crossing a different set of Malawi species (Cooper *et al.* 2011b). Such patterns are thought to confer a functional advantage in cichlids, with shorter jaws and steeper heads being useful for generating power during biting via increased mechanical advantage and resisting forces propagated through to upper jaws and to the skull (Cooper *et al.* 2011b; Wainwright *et al.* 2004).

The QTL associated with this axis all showed patterns of allelic additivity or dominance effects (4 of 4 cases), whereas many QTL for subsequent axes (9 of 13) (Table 3, Table 4) showed evidence of overdominance. This suggests that inheritance patterns may play a role in determining what loci contribute to specific axes of craniofacial change. In the context of evolvablity, traits that have an additive or dominant inheritance pattern could more readily provide a trajectory on which continuous divergence can proceed. This is because heterozygotes possess a phenotype that is intermediate to, or more often tending toward, a phenotype that matches a homozygote. If this trajectory confers the greatest fitness advantage, then this means that costs due to heterozygosity can be mitigated to some degree by keeping phenotypes on this trajectory, but with changing positions. Such a scenario matches our previous findings of largely continuous phenotypic distributions on a primary axis largely consisting of jaw length variation for each of the African cichlid radiations (Cooper *et al.* 2010).

Overdominant inheritance patterns, however, may evolve less readily, and thus associated loci should contribute less variation overall to adaptive phenotypic divergence in cichlids. This is because in cases where overdominance produces an adaptive phenotype it would require selection to maintain a balanced polymorphism (Richman 2003). If another locus affects the same phenotype through additive or dominant patterns of inheritance, then it should be a more efficient target of selection and is more likely to be maintained over time. Alternatively, in cases where overdominant phenotypes produce a maladaptive phenotype, hybrids will be at a disadvantage, which could potentially lead to assortative mating, restricted gene flow, and the reinforcement of disruptive natural selection. We speculate that the sorting of craniofacial loci with additive/dominant effects may underlie patterns of continuous morphological variation within and across species in the lake, whereas the sorting of loci with overdominant effects may facilitate craniofacial divergence between closely related species.

### Candidate loci for interspecific craniofacial shape variation

Combining traditional QTL mapping with genome-wide scans for divergent loci has proven to be an efficient means of implicating the specific genes that underlie QTL intervals (Rogers and Bernatchez 2007; Cadic *et al.* 2013; Roesti *et al.* 2014; Albertson *et al.* 2014). The utility of such an approach is especially high for systems that exhibit high levels of phenotypic divergence and low levels of genomic divergence, but are less tractable with respect to generating large hybrid populations for pedigree mapping (*e.g.*, they are large, aggressive, and/or have long generation times).

The power of the approach is limited by the size of the C.I. of the QTL. We therefore discuss data for a select set of QTL that map to relatively small genetic intervals, which correspond to discrete physical scaffolds. We consider SNPs to exhibit a signature of divergence when F_ST_ ≥0.57, an empirical threshold for divergence between cichlid genera (Mims *et al.* 2010), but most of the strongest candidates had F_ST_ values close to 1.00. Given the recent origin and rapid diversification of Lake Malawi cichlids (Turner *et al.* 2001), as well as ongoing gene flow between lineages (Mims *et al.* 2010), it is highly unlikely that such outlier loci have arisen due to genetic drift or other neutral processes (Loh *et al.* 2008). These data are presented in Table S2. Among autosomal loci, the QTL for RW1 that maps to LG3 (5.0 cM–23.5 cM) (Table 3) is anchored to four relatively short physical scaffolds that correspond to just under 5 Mb of sequence. Our genome scan identified 18 SNPs with outlier F_ST_ values (approximately 1 every 277 kb). Notably, four of these were adjacent to the gene *serpinh1*, which encodes Hsp47, a collagen-specific chaperone (Widmer *et al.* 2012) that is expressed in cartilage precursor cells in zebrafish (Lele *et al.* 1997). The QTL for RW3 that maps to LG10.1 (0.0 cM–5.0 cM) (Table 3) is anchored to a single physical scaffold that is ∼7 Mb long. We identified 15 SNPs with high F_ST_ values (1/467 kb) on this scaffold, including one located 5′ of *frem2a*. This gene is expressed in the developing pharyngeal arches in zebrafish (Gautier *et al.* 2008) and underlies certain forms of Fraser syndrome in humans (OMIM: 21900), which is characterized by a range of skeletal and craniofacial defects. A second QTL for RW3 maps to LG19, which is anchored to three scaffolds and just under 9 Mb of sequence. We identified 60 SNPs with high F_ST_ values (1/150 kb) on this scaffold, several of which are around two genes that are noteworthy with respect to variation in skull shape. One exonic (synonymous) and two intronic SNPs with divergent F_ST_ values were uncovered within the gene *sec23a*. This gene is mutated in humans with craniolenticulosutural dysplasia (OMIM: 607812), which is characterized by a suite of craniofacial dysmorphologies. It is also the mutated gene in the zebrafish "crusher" mutant, which has a small and malformed head skeleton (Lang *et al.* 2006). A second strong candidate gene within this interval is *runx2a*, for which two divergent SNPs were noted, one intronic (F_ST_ = 1.00) and one 5′ (F_ST_ = 1.00). Cleidocranial dysplasia in humans (OMIM: 119600) is caused by haploinsufficiency of this gene, which includes several minor craniofacial dysmorphologies.

It is notable that for these candidate genes the LF allele was evolutionarily derived—*e.g.*, TRC, *M. zebra*, *P. nyererei*, *A. burtoni*, *N. brichardi*, *and O. niloticus* were all found to carry the opposite allele (cichlid genomes can be found at http://em-x1.gurdon.cam.ac.uk/). This trend is consistent with LF expressing a highly derived craniofacial phenotype relative to other cichlids. The exception to this trend was in *runx2a* where the LF allele is derived for the 5′ SNP, but the TRC allele is derived for the intronic SNP. Because both TRC and LF possess relatively short “faces” compared to other cichlids, it is possible that separate polymorphisms in *runx2a* underlie the development of this phenotype in these two lineages. This hypothesis is consistent with allelic effects at this QTL, where heterozygous animals have the lowest scores on average compared to both homozygous genotypes (RW3 LG19) (Table 3).

### Sexual dimorphism in shape: implications for sexual conflict and ecomorphological divergence in a nested trait

We suggest that sexual dimorphism in craniofacial shape has contributed to shape variation in the adaptive radiation of Malawi as a whole. Sexual dimorphism was prevalent in our F_2_ data and was similar to the primary axis of variation for the Malawi radiation (Figure 2). Also, sex was found to have a significant effect on the primary axis of shape (*i.e.*, RW1) that had a shared trajectory with the radiation. Male F_2_s tended to have a steeper profile relative to females, which should confer an advantage for biting. Given that rock-dwelling adult male cichlids tend to hold territories along the rocky shoreline, there is increased opportunity to forage from the substrate with a biting mode (Ribbink *et al.* 1983; Konings 2001). In addition, LF and TRC males are highly territorial, especially toward conspecific males, and frequently engage in aggressive interactions that involve the locking of jaws and reciprocal biting of flanks (Ribbink *et al.* 1983; Konings 2001; R. C. Albertson, unpublished data). All other things being equal (*e.g.*, levels of aggression, size, etc.), males with a more extreme “biting” morphology would have a competitive advantage during this behavior. Alternatively, females of both LF and TRC are nonterritorial and spend more time in the water column, surveying males and searching for suitable foraging sites (Ribbink *et al.* 1983; Konings 2001). Thus, there should be more opportunity for females to encounter prey in the water column; a longer, shallower profile would facilitate this mode of feeding.

### Candidate loci for sexually dimorphic craniofacial shape variation

Our test for QTL interactions shows that sex-specific shape is affected by the interaction between autosomal and sex-determining loci, and that the LF allele at the autosomal QTL on LG11 (90.0 cM–105.07 cM) results in a more extreme male-like phenotype. This ∼15-cM interval is anchored to three scaffolds and ∼8 Mb of sequence. We identified 57 divergent SNPs within this interval (1/140 kb), including several strong candidates with respect to bone formation and metabolism (Table S2). First, a divergent SNP (F_ST_ = 0.95) was identified within the exon (synonymous mutation) of the gene *slc39a1*, overexpression of which has been shown to be sufficient to induce osteoblast differentiation in mesenchymal stem cells (Tang *et al.* 2006). It is also expressed in osteoclasts (Khadeer *et al.* 2005). Another divergent SNP (F_ST_ = 0.812) was identified 5′ of a second *solute carrier family* member, *slc4a7*. This gene is expressed in osteoclasts, and osteoclast degradation of hydroxyapatite during bone remodeling requires *slc4a7* activity (Riihonen *et al.* 2010). A divergent intronic SNP (F_ST_ = 1.00) was also found within the gene *ADAM15*, which negatively regulates osteoblast proliferation and function by inhibiting nuclear translocation of β-catenin within osteoblasts (Marzia *et al.* 2011). Finally, three divergent SNPs were found within and around the gene *tsp3* (one intronic SNP F_ST_ = 1.00, two 3′ SNPs F_ST_ = 1.00 and 0.814). Mice lacking *Tsp3* show normal prenatal skeletal patterning but display subtle abnormalities in the developing postnatal skeleton, which suggests that this gene plays a role in the regulation of skeletal growth in mice (Hankenson *et al.* 2005).

### Sex-linked or sex-influenced craniofacial shape? Insights from candidate loci

We have demonstrated that sex has a measurable effect on craniofacial shape that appears to be ecologically relevant. We further show that the genetic basis for this sexual dimorphism is both autosomal and associated with the major sex-determining locus. However, our genetic data cannot discriminate between whether these effects are the result of sex-linked craniofacial loci or sex-influenced craniofacial development. Given that Malawi cichlids lack sex chromosomes, sex linkage seems less likely. Moreover, our mapping of DF1 (sex) provides some support for the idea that craniofacial development is sex-influenced. The autosomal QTL for DF1 that maps to LG11 (90.0 cM–105.07 cM) is particularly interesting and may ultimately offer mechanistic insights into how sex-influenced development may occur. Given that bone metabolism can be significantly affected by sex hormones (Khosla *et al.* 2012; Ferlin *et al.* 2013), and that this locus interacts with the sex determination locus, we might expect genes involved in osteoblast/osteoclast differentiation/activity to underlie this QTL. Notably, the candidates described above (*e.g.*, *slc39a1*, *ADAM15*) for this locus represent such factors. For example, not only does *ADAM15* play important roles in osteoblast development (Marzia *et al.* 2011) but also its expression is influenced by estrogen levels (Nadadur *et al.* 1985). In addition, circulating transcript numbers of *slc 39a1* respond to growth hormone levels *in vivo* (Sun *et al.* 2013). Further analysis of this locus within the context of sex may have the potential to provide new mechanistic insights into how sex influences shape.

Although sex-influenced craniofacial development may be the most likely explanation for the trends observed here, we cannot rule out the possibility of sex linkage. Physical linkage between sex determination and pigmentation has between documented for Lake Malawi cichlids (Roberts *et al.* 2009) on LG5. It is therefore at least possible that craniofacial loci are physically linked to sex determination on LG7. In this cross, sex determination maps to a narrow interval that is anchored to two physical scaffolds, MZ scaffolds 0 and 21. Unfortunately, our genome scan included both males and females of each species; therefore, we cannot use divergent loci to implicate causative genes at the sex-determining locus. Nevertheless, it is tempting to speculate that one or more of a few well-known craniofacial genes located on these scaffolds might play a role in sex-linked craniofacial shape variation (Table S2). For example, *col11a1b* is on scaffold 21, and the homolog is responsible for both Stickler (OMIM: 108300) and Marshall (OMIM: 154780) syndromes in humans, which are characterized by an array of craniofacial abnormalities. In addition, the adjacent genes *tbx3a* and *tbx5a* are on scaffold 0, which play critical roles in skeletal development and homeostasis (Govoni *et al.* 2009; Yao *et al.* 2014), and are implicated in human skeletal malformations (*e.g.*, ulnar-mammary syndrome, OMIM: 181450; Holt-Oram syndrome, OMIM: 142900). Unfortunately, neither the specific gene nor the extent of linkage disequalibrium has been determined for sex specification on LG7, and without this information we cannot say whether these candidates fall within this interval. Nevertheless, it is notable that genes with known and pronounced influences on craniofacial development and bone metabolism are within the QTL interval for sex determination.

## Conclusions

Given our findings, we suggest that nested variation, especially in the form of ecological sexual dimorphism, should be considered in greater detail for cases of adaptive divergence. Theories regarding the origins of adaptive radiations typically suggest phenotypic divergence for the purpose of resource use as a first step (Skulason and Smith 1995). These changes in phenotype can have a genetic basis but can also incorporate environmental inputs that enhance phenotypic differences through changes in development (*i.e.*, phenotypic plasticity). Our data suggest that in some cases divergence can also be promoted through sexual dimorphism in ecologically relevant characters. The reduction in competition between sexes afforded by such dimorphism could represent a robust evolutionarily stable strategy.

No matter the utility of such a dimorphism, however, theory also predicts an evolutionary limit to phenotypic divergence between the sexes because they are obligated to interbreed. In sticklebacks where sexual dimorphism in shape can exceed the degree of ecological divergence between ecomorphs (Cooper *et al.* 2011a), we may be witnessing an extreme upper limit of ecological sexual dimorphism. The ecological community of fresh water stickleback is relatively simple, with no close heterospecific vertebrate competitors for resources (but see Gray *et al.* 2005). Thus, sexual dimorphism in ecologically relevant shape should be free to push the limits of what is possible given the constants of genetic architecture. Alternatively, lacustrine cichlid species often exist within complex communities, surrounded by close ecological competitors (Ribbink *et al.* 1983; Konings 2001). In such systems, ecological sexual dimorphism should be more constrained as females with more pelagic-like head shapes compete for resources with obligate pelagic species. Thus, ecological sexual dimorphism that is observed in older and/or more complex radiations is likely to be more subtle than that seen in younger, less complex radiations. Nevertheless, this nested pattern deserves more study because it may represent an important mechanism at the root of both the origins and the maintenance of biodiversity.

## 

## Supplementary Material

Supporting Information
