# Supplementary material for: Nested Levels of Adaptive Divergence: The Genetic Basis of Craniofacial Divergence and Ecological Sexual Dimorphism
Source: G3 (Bethesda). 2015 Jun 1;5(8):1613–24. doi: 10.1534/g3.115.018226 (PMC4528318; doi:10.1534/g3.115.018226)
Supplement: Supporting Information [file supp_5_8_1613__index.html]

Nested Levels of Adaptive Divergence: The Genetic Basis of Craniofacial Divergence and Ecological Sexual Dimorphism — Supporting Information 

# Nested Levels of Adaptive Divergence: The Genetic Basis of Craniofacial Divergence and Ecological Sexual Dimorphism

## Supporting Information for Parsons *et al.*, 2015

**Files in this Data Supplement:**

- Table S1 - Results of an initial genome scan for morphological QTL using models that include sex as a covariate, and models without sex included. (PDF, 220 KB)
- Table S2 - Candidate loci associated with craniofacial shape. (.xlsx, 53 KB)
