# Supplementary material for: Nested Levels of Adaptive Divergence: The Genetic Basis of Craniofacial Divergence and Ecological Sexual Dimorphism
Source: G3 (Bethesda). 2015 Jun 1;5(8):1613–24. doi: 10.1534/g3.115.018226 (PMC4528318; doi:10.1534/g3.115.018226)
Supplement: Supporting Information [file supp_g3.115.018226_018226SI.pdf]

G3 · Genes | Genomes | Genetics

Nested Levels of Adaptive Divergence: The Genetic Basis of Craniofacial Divergence and Ecological Sexual Dimorphism

Kevin J. Parsons[1], Jason Wang[2], Graeme Anderson[1], R. Craig Albertson[2]

[1] Institute of Biodiversity, Animal Health & Comparative Medicine, University of Glasgow, Glasgow G12 8QQ

[2] Department of Biology, 221 Morrill Science Center, University of Massachusetts, Amherst MA 01003

Corresponding author: Kevin Parsons (Kevin.Parsons@glasgow.ac.uk)

DOI: 10.1534/g3.115.018226

Table S1 Results of an initial genome scan for morphological QTL using models that include sex as a covariate, and models without sex included. Abbreviations are as follows: LG=linkage group, pos=position, add=additive effects, dom=dominance effect, PVE = percent variance explained. The effect of alleles on the traits (principal component scores) are given, with A alleles coming from Labeotropheus fuelleborni, and B alleles from Tropheops 'red cheek'.

Trait	LOD	LG	pos	marker	AA	AB	BB	add	dom	PVE
Including sex as a covariate										
PC1	3.54	1	35.0	c7.3954257	0.00433	0.00004	-0.00536	0.00484	0.00054	7.5
	3.48	13	0.0	c26.2061138	-0.00844	0.00154	0.00400	-0.00622	0.00299	7.3
	3.26	13	2.5	c26.802414	-0.00919	0.00156	0.00317	-0.00618	0.00379	6.9
	3.93	19	0.0	c96.185530	-0.00614	-0.00112	0.01145	-0.00879	-0.00322	8.3
PC2	3.13	7	36.3	c0.7061265	-0.01216	0.00137	0.00779	-0.00997	0.00287	6.6
	3.17	24	2.7	c106.1399010	0.00354	0.00089	-0.00944	0.00649	0.00339	6.7
PC3	3.01	17	41.0	c17.3331825	-0.00169	0.00142	-0.00190	0.00010	0.00251	6.4
	3.28	23	44.0	c82.1205886	-0.00101	-0.00073	0.00232	-0.00167	-0.00102	6.9
	4.14	13	9.0	c26.2173503	0.00081	-0.00033	-0.00114	0.00097	-8.20E-07	8.7
	4.24	13	0.0	c26.1362137	0.00142	-0.00104	-0.00021	0.00082	-0.00112	8.9

PC4	4.43	7	28.0	c21.2195347	-0.00310	-0.00029	0.00273	-0.00291	0.00004	9.2
	3.70	24	28.0	c132.348543	0.00014	-0.00117	0.00093	-0.00039	-0.00112	7.8
PC5	4.12	15	26.8	c146.88596	0.00194	0.00020	-0.00113	0.00153	-0.00031	8.6
	3.69	5	61.0	c10.6870455	0.00508	-0.00148	-0.00083	0.00296	-0.00287	7.8
	3.67	2	52.7	c11.8641264	0.00536	0.00011	-0.00340	0.00438	-0.00092	7.7
	3.74	10	1.8	c22.4015123	0.00484	-0.00097	-0.00149	0.00317	-0.00216	7.9

Not including sex as a covariate

PC1	5.68	7	14.6	c124.1667602	-0.007565333	-0.00260	0.01262	-0.01009	-0.00383	11.7
	3.20	19	0.0	c96.185530	-0.00621162	-0.00104	0.01134	-0.00877	-0.00308	6.8
PC2	7.08	7	30.0	c0.5071706	-0.011902264	0.00172	0.00875	-0.01033	0.00244	14.4
PC3	3.00	13	32.4	c62.2721053	-4.97E-03	-9.21E-05	4.84E-03	-0.00490	2.07E-05	6.4
sex	27.56	7	26.0	21.2195347	0.000555096	0.00045	-0.00088	0.00072	0.000386	45.3
